# Editorial: Novel CNS targeting molecules, methods, and therapeutics in multiple sclerosis

**DOI:** 10.3389/fimmu.2025.1699850

**Published:** 2025-09-18

**Authors:** Rune Alexander Aamodt Høglund, Stella E. Tsirka, Makoto Inoue

**Affiliations:** ^1^ Department of Neurology, Akershus University Hospital, Lørenskog, Norway; ^2^ Department of Pharmacological Sciences, Renaissance School of Medicine at Stony Brook University, Stony Brook, NY, United States; ^3^ Department of Comparative Biosciences, College of Veterinary Medicine, University of Illinois at Urbana-Champaign, Champaign, IL, United States

**Keywords:** CNS therapeutics, diagnostic method, biomarker, neurogenesis, remyelination, neuronal progenitor cells, oligodedrocyte progenitor cells

Multiple sclerosis (MS) is a chronic autoimmune and neurodegenerative disease of the central nervous system (CNS), affecting nearly three million people worldwide. Understanding the progression of pathogenic alterations in the CNS during MS is critical for identifying therapeutic targets and improving diagnostic approaches. Novel drugs aimed at CNS pathology hold promise for enhancing treatment efficacy and reducing adverse effects. This Research Topic highlights recent advances in MS autoimmunity, with the goal of showcasing the development of targeted therapies and diagnostic strategies.

Chen et al. investigated the relationship between microglial activation and remyelination status in MS. Microglia play a dual role: they facilitate clearance of myelin debris and promote the recruitment of oligodendrocyte precursor cells (OPCs) to lesion sites, but they also contribute to neuroinflammation, death of oligodendrocytes, and neuronal damage. The authors compared microglial subtypes and early-stage oligodendrocytes in remyelinated regions of MS patients, identifying differences between efficiently and poorly remyelinating individuals.

Hoffrogge et al. reported that antagonism of the purinergic P2X7 receptor (P2X7R) ameliorates experimental autoimmune encephalomyelitis (EAE) by suppressing microglial activation without affecting the peripheral immune system. P2X7R, a ligand-gated ion channel activated by extracellular ATP, is strongly upregulated in reactive astrocytes and microglia within MS lesions. Sustained P2X7R activation contributes to lesion formation, suggesting that P2X7R antagonists hold therapeutic potential in MS.

Sindi et al. demonstrated that positive allosteric modulators of AMPA-type glutamate receptors (AMPA-PAMs) protect against demyelination in the EAE and cuprizone models. AMPA-PAMs enhance synaptic transmission without causing excitotoxicity and exhibit neuroprotective effects. Notably, they increase OPC numbers, suggesting their potential in promoting remyelination.

Neupokoeva et al. proposed a novel diagnostic method for MS using blood serum surface-enhanced Raman scattering (SERS) combined with machine learning analysis. Compared to MRI, the current gold standard for detecting CNS demyelination, but with practical limitations, SERS offers exceptionally high specificity and sensitivity for diagnosing MS patients.

Anandan et al. reviewed the potential of brain-derived blood biomarkers in MS. The authors provide a comprehensive summary of all biomarkers of the past, present, and future, including brain-derived extracellular vesicles, which may serve as valuable tools for diagnosis, prognosis, monitoring, and personalized treatment.

Altogether, this Research Topic advances our understanding of CNS-targeted therapies and diagnostic innovations in MS, helping to shape future research agendas toward improving patient care.

